# Adherence to sleep recommendations is associated with higher satisfaction with life among Norwegian adolescents

**DOI:** 10.1186/s12889-024-18725-1

**Published:** 2024-05-10

**Authors:** Erik Grasaas, Sergej Ostojic, Henriette Jahre

**Affiliations:** 1 Department of Nutrition and Public Health, Faculty of Health and Sport Sciences, University in Agder, Kristiansand, Postbox 422, 4604 Norway; 2 Department of Rehabilitation Science and Health Technology, Center for Intelligent Musculoskeletal health, Oslo Metropolitan University, Oslo, Norway

**Keywords:** Adolescents, Sleep, Satisfaction with life, Quality of life

## Abstract

**Background:**

Sleep plays a crucial role in the health and well-being of adolescents; however, inadequate sleep is frequently reported in numerous countries. This current paper aimed to describe sleep duration, factors impacting sleep, consequences of insufficient sleep and satisfaction with life in Norwegian adolescents, stratified by sex and by adherence to the 8-hour sleep recommendation, and to examine potential associations between adherence to the 8-hours sleep recommendation and satisfaction with life.

**Methods:**

This is a cross-sectional study using data from the Norwegian Ungdata Survey, collected in 2021. Adolescents from five Norwegian counties were included, comprising a total of 32,161 upper secondary school students. Study variables were collected through an electronic questionnaire administered during school hours and all data are anonymous. Descriptive data of sleep patterns are presented, and linear regressions were conducted adjusting for SES, perceived stress, physical activity level, over-the-counter analgesics use, grade level and screen time.

**Results:**

73% of adolescents did not adhere to the 8-hours of sleep recommendation per night, with similar results for girls and boys. 64% reported tiredness at school (minimum 1–2 days weekly) and 62% reported that screen time negatively affected their ability to sleep. 23% reported that gaming affected their sleep, with a higher prevalence in boys than girls. Satisfaction with life score was 7.0 ± 1.9 points (out of 10) for the total sample, with higher scores for boys (7.3 ± 1.8 points) than girls (6.9 ± 1.9 points). Regressions revealed a positive association with satisfaction with life (B = 0.31, 95% [0.15 to 0.48]) in adolescents adhering to sleep recommendation of 8h compared to the ones not adhering to the sleep recommendation.

**Conclusions:**

Most Norwegian adolescents fail to adhere to the 8-hours of sleep recommendation and the majority feel tired at school or during activities. More than half of adolescents reported that screen time negatively affected their ability to sleep. Adhering to the sleep recommendation was associated with higher life satisfaction. Our findings highlight the importance of sufficient sleep in adolescents, while future research is needed to examine other sleep related measures on adolescents´ satisfaction with life.

**Supplementary Information:**

The online version contains supplementary material available at 10.1186/s12889-024-18725-1.

## Background

Sleep is recognized as a crucial factor for children’s and adolescents’ health and wellbeing [1]. Sleep recommendation vary with age and according to the US National Sleep Foundation teenagers are recommended 8–10 h of sleep [2]. However, when Gariepy and colleagues examined sleep patterns in 24 European and North American Countries, including 165,793 adolescents, findings revealed that insufficient sleep is prevalent in many countries [3]. Insufficient sleep impacts the daytime functioning in adolescents, leading to various negative consequences in their lives [4]. Extensive research evidence has reported that insufficient sleep among adolescents increases the risk of physical, psychosocial, and behavioral problems, and is associated with worse health outcomes [4–9].

When examining sleep duration in adolescents, research evidence refers to both the time in bed (TIB) and the sleep onset time (SOT) until wakening as estimates of sleep duration. It is suggested that TIB might overestimate the sleep duration in adolescence [10], as adolescents don’t immediately fall asleep when they go to bed. The latency time from going to bed to SOT was reported to be on average around 17 min for older adolescents in 2002 [11]. However, considering the commonality of screen time use before bedtime nowadays, it is presumed that this average time has increased [12, 13]. A recent Norwegian sleep study reported the average time between going to bed and SOT was over one hour, revealing that eight in ten adolescents in upper secondary school actually failed to obtain the minimum recommended amount of sleep (8 h) on school days [10].

Research evidence points to several causes of insufficient sleep in adolescence, which are commonly categorized into internal- and external factors. External factors may include reduced parental involvement, excessive homework or activities, perceived stress, and screen time usage, whereas internal factors refer to puberty and biological processes such as a shift in the circadian rhythm [4, 14–18]. Regardless of its causes, insufficient sleep is reported to impact all aspects of adolescents’ daily life and wellbeing [4–7, 9, 14, 16, 17, 19–21]. A well-known indicator of subjective well-being is Life Satisfaction measure, which serves as a useful complement for comparing data across ages and countries, and is assessed to evaluate their life as a whole rather than their current feelings [22]. Satisfaction with life is therefore a well-known measure to indicate happiness across countries and time [22]. According to Diener, the measure reflects the cognitive judgment of one´s satisfaction with life [23]. It has been reported that girls tend to report lower satisfaction with life compared to boys during adolescence, along with a general decrease in satisfaction with life throughout this period [24].

Since most Norwegian adolescents do not meet the recommended 8 h of sleep [10], it is crucial to investigate potential consequences for this age group. Since life satisfactions is a good indicator of adolescent’s well-being, and a proxy for happiness it would be interesting to investigate the relationship between sleep duration and satisfaction with life using large dataset with high response rate. Such research can provide substantial insights for both practice and policy development, potentially emphasizing the significance of adhering to the sleep recommendations in Norway. The main aims of the present study were (1) to describe sleep duration, factors impacting sleep, consequences of insufficient sleep and satisfaction with life in Norwegian adolescents, stratified by sex and by adherence to the 8-hour sleep recommendation, and (2) to examine potential association between adherence to the 8-hours of sleep recommendation and satisfaction with life in Norwegian adolescents.

We hypothesized that adolescents adhering to the 8-hour sleep recommendation would have a more positive association to satisfaction with life compared to adolescents sleeping seven hours or less.

## Methods

This study is reported according to the Strengthening the Reporting of Observational Studies in Epidemiology (STROBE) guidelines [25].

### Study design

This is a cross-sectional study using data from the Norwegian Ungdata Survey, collected in 2021. Ungdata is conducted by Norwegian Social Research (NOVA) at Oslo Metropolitan University in collaboration with regional center for drug rehabilitation (KoRus) and the municipal sector’s organization (KS). It is a quality-assured system for carrying out repeated national surveys among pupils in lower and upper secondary schools related to all aspects of health and wellbeing [26].

The Ungdata survey includes adolescents from lower and upper secondary schools from almost all municipalities in Norway. The survey consists of a comprehensive electronic questionnaire, with a mandatory basic module for all the municipalities, and a set of optional, predefined questions, which municipalities and counties can choose from. In addition, self-composed questions may also be added by the municipalities, counties or collaborating universities. The Ungdata project is financed from the national budget through grants from the Norwegian Directorate of Health [26].

Ungdata is a free survey offered to all Norwegian counties and their respective municipalities. The yearly sampling is administered by including specific counties. Within the next two following years, the rest of the counties are recruited. According to Ungdata, within a three-year period, close to all Norwegian municipalities have participated in the survey [27]. Therefore, the national presented findings from Ungdata usually comprises data from the last three years, which results in a representative study sample for the whole target population. However, according to Ungdata, the survey from 2021 should be assessed more separately, due to the pandemic and due to the record high participation of municipalities this year [27]. In the supplementary information material provided by Ungdata, there are coding for different counties, municipalities, and schools. Indicating that schools were the primary sampling unit. However, the Ungdata dataset does not include a variable including the separate schools.

### Study setting

The surveys take place during one school hour (45–55 min) and are carried out electronically by the respective teacher. Pupils who are not interested in taking the survey are provided other schoolwork. The research evidence extracted from the Ungdata Survey is well suited for planning and initiating work towards adolescents and public health [26].

### Participants

Norwegian adolescents from upper secondary school (16–19 years of age) are included in this study. The response rate was 67% from the whole country [27]. Adolescents from five counties (*n* = 32,161) are included in this specific study because they were the only counties that included sleep in their questionnaire (optional question). Number of participants were lower in questions regarding screentime/gaming affecting their sleep, as these questions were included in only two and three counties, respectively.

### Variables

#### Exposure: sleep

Sleep duration was measured using the question “*How many hours of sleep did you get last night”.* Seven response alternatives were provided, ranging from, 6 h or less or hourly up to 12 h or more. These response alternatives were recoded into a dichotomous variable to determine whether participants met (8 h or more) or did not meet (7 h or less) the international recommendations for sleep in adolescents [2]. Problems falling asleep and being tired in school or in activities was measured using four response alternatives, “no days”, “1–2 days”, “3–4 days” and “5 days or more”. If screentime or gaming affected their sleep was measured with two response alternatives, “yes” or “no”. These questions were formulated as: Has screentime affected you to not getting enough sleep and has gaming affected you to not getting enough sleep?

#### Outcome: satisfaction with life

Satisfaction with life was assessed using the question: “On *a scale from 0 to10, how happy are you with your life these days?”* Higher scores indicated greater satisfaction with life. This question on satisfaction with life was originally employed in a large Norwegian study called “Young in Oslo in 2018” [28], including 25,348 adolescents. Using a single-item measure for satisfaction with life has across samples demonstrated a substantial degree of validity and performed similar to the multiple-item satisfaction with life scale [29]. Especially in adolescence, its reported that a single-item life satisfaction measures perform as well the satisfaction with life scale [30].

#### Demographic variables and covariates

The Ungdata study includes demographic measures such as gender, grade level, respective county and municipality, and measures of socioeconomic status (SES). SES is measured by several questions related to parental educational level, books in their home and their level of prosperity. A total sum is calculated based on these three categories and recoded into values from 0 to 3, off which 0 represent lowest SES and 3 the highest SES [31]. This measure is reported as a validated construct of SES [26]. As the Ungdata Survey is anonymous, data on age is not available. For overview of study variables and response rate, see Table 1.

**Table 1:** List of study variables and corresponding response rate

Variable	*N*	Response rate %
Sleep duration	32,055	99.7%
Problems falling asleep	29,741	92.5%
Being tired in school or in activities	31,558	98.1%
Screen time affecting not getting enough sleep	2,114	99.8%
Gaming affecting not getting enough sleep	15,936	99.8%
Satisfaction with life SES Perceived stress Physical activity OTCA	31,960 32,089 30,386 31,819 31,850	99.4% 99.9% 94.5% 98.9% 99.0%

Perceived stress level, physical activity level and use of over-the-counter analgesics (OTCA) are included as categorical covariates in the regression analysis [26].

Perceived stress level was measured by using the question “*Have you experience so much pressure the last week that you had problems managing it?”*. Four response alternatives were provided, “not at all”, “to a small degree”, “to a large degree” and “to a very large degree” [26]. Perceived stress was found as a relevant psychological covariate in Norwegian adolescents due to the link to exposure and outcome [32, 33].

Physical activity level was measured using the question “*How often are you so physical active that you become short of breath or sweaty?*” Six response alternatives were provided from “rare”, to different times a week, up to “at least 5 times a week” [26].

The use over OTCA was measured by using the question *“How often have you used non-prescription drugs (Paracet, Ibux and similar) during the last month?”*. Five response alternatives were provided ranging from “no times”, different times a week to “daily” [26].

### Ethical consideration

Participation in the Ungdata survey is voluntary and informed written consent were provided by the adolescents. All questions from Ungdata included in this current study is approved by the Norwegian Agency for Shared Services in Education and Research (ref. 821,474), known as SIKT [34]. As the survey is conducted in May-June, adolescents in upper secondary school were 16 years or older and did not need parental consent. The study is conducted in accordance with the Helsinki Declaration.

### Statistical analyses

All statistical analyses were conducted using IBM SPSS Statistics for Windows, Version 25.0 (IBM Corp., Armonk, NY, USA). For the descriptive measures, continuous variables are described using means and standard deviations (SDs), and categorical variables are presented with counts and percentages. Sleep variables are presented for the total study sample and stratified into girls and boys, and into those who achieved the recommended sleep duration or not. Linear regressions analyses were conducted to examine the association between achieving the recommended sleep duration (8 h or more) or not and satisfaction with life. Stratified regressions analyses for girls and boys were conducted to investigate potential sex differences in the associations. One sample proportion test revealed high precision (CI) in estimates across the descriptive study variables. Both crude and multiple regression analysis adjusted for SES, perceived stress, physical activity level, OTCA use, grade level and screen time are presented. The results are presented with beta coefficients with 95% confidence intervals and R-squared (R^2^). *P*-values < 0.05 were considered statistically significant, and all tests were two‐sided. Sensitivity analysis using 7-hours as a cut-off were used to check the robustness of the results. Due to the large sample size and relatively small number of missing, no imputation or bootstrapping was considered necessary.

## Results

### Participants

In total, 32,161 adolescents from five Counties in Norway were included in the analyses. Response rate remained high in selected study variables ranging from 92.5 to 99.9% (Table 1). More boys than girls participated (53% versus 47%), 42% of the participants were from 1st grade, 35% from 2nd grade, and 23% from 3rd grade (Table 2).

**Table 2:** Sample characteristics (*n* = 32,161)

	*N* (%)
**County** More and Romsdal Innlandet Vestfold and Telemark Vestland Trøndelag	137 (0.4%) 7,764 (24.1%) 8,962 (27.9%) 11,819 (36.7%) 3,479 (10.8%)
**Sex** ^**a**^ Girls Boys	16,895 (53.2%) 14,841 (46.8%)
**Grade level** ^**b**^ 1st grades 2nd grades 3rd grades	13,389 (41.8%) 11,196 (34.9%) 7,469 (23.3%)

^a^*N* = 31,736; ^b^*N* = 32,055

### Descriptive data of sleep in Norwegian adolescents

Descriptive data of sleep variables are presented in Table 3. 73% of adolescents did not adhere to the 8-hours of sleep recommendation, with similar results for girls and boys. 62% of respondents reported experiencing difficulties falling asleep on at least one day or more. This issue was more prevalent among girls (68%) than boys (56%). Feeling tired at school at least once a week was reported by 64%, by 71% of the girls and by 56% of the boys. 62% of participants stated that screen time negatively affected their ability to get enough sleep, 66% of girls and 57% of boys reported this. 23% of the adolescents reported that gaming affected their ability to get enough sleep, 11% of the girls and 38% of the boys. Satisfaction with life score was 7.0 ± 1.9 points for the total sample (Table 3), with higher scores for boys (7.3 ± 1.8 points) than in girls (6.9 ± 1.9 points) (Table 3).

**Table 3:** Characteristics of sleep variables

Study variable	All (*N* %)	Girls (*N* %)	Boys (*N* %)
**Sleep duration** ^**a**^			
6 hours or less	12,524 (39.1%)	6,600 (39.2%)	5,704 (38.5%)
7 hours	10,958 (34.2%)	5,793 (34.4%)	5,071 (34.3%)
8 hours	6,453 (20.1%)	3,366 (20.0%)	3,035 (20.5%)
9 hours	1,568 (4.9%)	828 (4.9%)	720 (4.9%)
10 hours	314 (1.0%)	162 (1.0%)	148 (1.0%)
11 hours	102 (0.3%)	50 (0.3%)	48 (0.3%)
12 hours or more	136 (0.4%)	44 (0.3%)	75 (4.1%)
**Problems falling asleep** ^**b**^			
No days	11,159 (37.5%)	5,016 (32.1%)	6,050 (44.1%)
1–2 days	10,441 (35.1%)	5,723 (36.6%)	4,615 (33.6%)
3–4 days	4690 (15.8%)	2,847 (18.2%)	1,766 (12.9%)
5 days or more	3,451 (11.6%)	2,043 (13.1%)	1,302 (9.5%)
**Being tired in school or in activities** ^**c**^			
No days	11,238 (35.6%)	4,784 (28.8%)	6,350 (43.6%)
1–2 days	11,673 (37.0%)	6,332 (38.1%)	5,207 (35.8%)
3–4 days	5,276 (16.7%)	3,401 (20.5%)	1,806 (12.4%)
5 days or more	3,371 (10.7%)	2,096 (12.6%)	1,186 (8.2%)
**Screen time affected not getting enough sleep** ^**d**^			
Yes	1,304 (61.7%)	747 (65.9%)	539 (56.7%)
No	810 (38.3%)	387 (34.1%)	411 (43.3%)
**Gaming affected not getting enough sleep** ^**e**^			
Yes	3,734 (23.4%)	900 (10.7%)	2,766 (37.8%)
No	12,202 (76.6%)	7,531 (89.3%)	4552 (63.6%)
**Satisfaction with life (0–10)(mean/SD)** ^**f**^	7.0 (1.9)	6.7 (1.9)	7.3 (1.8)

^**a**^*N* = 32,055, ^b^*N* = 29,741, ^c^*N* = 31,558, ^d^*N* = 2114 included in the county of Vestland and Trondelag, ^e^*N* = 15,936 included in the county of Vestland, Innlandet and Trondelag, ^f^*N* = 31,960

Descriptive measures stratified by adhereing to the 8-hours of sleep recommendations or not showed that 54% of adolescents receiving more than 8 h of sleep had no problems with falling asleep, while 32% of adolescents that did not achieve sleep recommendation had these struggles.

50% of adolescents adhering to the recommended sleep duration reported that they never felt tired at school or in other activities, whereas this was reported by 30% of those who did not adhere to the recommendations. Screen time was descriptively reported to affect sufficient sleep in 45% of those who met the recommendations, and in 67% of those who did not. Gaming was descriptively reported to affect sleep for 15% of those who slept 8 h or more, and 26% in those who slept less (Table 4).

**Table 4:** Characteristics of study variables stratified by recommendation of 8 h of sleep or not

Study variable	8 hours of sleep or more (N%)	7 hours of sleep or less (N%)
**Problems falling asleep**		
No days	4,298 (53.9%)	6,833 (31.5%)
1–2 days	2,827 (35.4%)	7,597 (35.0%)
3–4 days	620 (7.8%)	4,061 (18.7%)
5 days or more	233 (2.9%)	3,206 (14.8%)
**Being tired in school or in activities**		
No days	4,229 (50.3%)	6,983 (30.2%)
1–2 days	2,997 (35.6%)	8,663 (37.5%)
3–4 days	820 (9.8%)	4,448 (19.3%)
5 days or more	364 (4.3%)	2,996 (13.0%)
**Screen time affected sufficient sleep**		
Yes	223 (45.1%)	1,076 (66.8%)
No	272 (54.9%)	534 (33.2%)
**Gaming affected sufficient sleep**		
Yes	647 (15%)	3,071 (26.2%)
No	3,522 (84.5%)	8,661 (73.8%)
**Satisfaction with life (0–10) (mean/SD)**	7.5 (1.7)	6.9 (1.9)

### Associations between adhering to sleep recommendation or not on satisfaction with life

Adjusted multiple regression analysis stratified by sex showed that adhering to the recommended 8 h of sleep was positively associated with satisfaction with life in girls (B = 0.33; 95% CI [0.11–0.56]) and in boys (B = 0.27; 95% CI [0.02–0.52]) compared to those who did not adhere to the sleep recommendation (Table 5).

**Table 5:** Crude and multiple regressions between adhering to sleep recommendation or not (independent variable) on satisfaction with life (dependent variable) stratified by sex

Girls								Boys							
Unadjusted					Adjusted			Unadjusted				Adjusted			
**B**	**95% CI**	***p-*** **value**	**R** ^**2**^	***B***	**95% CI**	***P-value***	**R** ^**2**^	***B***	**95% CI**	***p-*** **value**	**R** ^**2**^	**B**	**95% CI**	**p-value**	R^2^
**0.66**															
	**0.60–0.73**	**< 0.001**	0.03	**0.33**	**0.11–0.56**	**0.003**	0.23	**0.58**	**0.52–0.65**	**< 0.001**	0.02	**0.27**	**0.02–0.52**	0.035	**0.16**

^Adjusted for: Socioeconomic status, perceived stress, physical activity level, over the counter analgesics, grade level and screen time

Crude regression analyses revealed a positive association between adhering to the 8-hours of sleep recommendation and satisfaction with life (B = 0.64; 95% CI [0.59–0.68]). Adjusted multiple regression analyses remained significant after adjusting for SES, perceived stress, physical activity level, OTCA use, grade level, screen time and sex (Table 6).

**Table 6:** Crude and multiple regressions between adhering to sleep recommendation or not (independent variable) on satisfaction with life (dependent variable)

		Unadjusted				Adjusted					
B	95% CI		*p-*value	R^2^	B		95% CI	*p-*value		R^2^	
0.64	0.59 to 0.68		*< 0.001*	*0.02*	*0.31*		*0.15–0.48*	*< 0.001*		*0.22*	

^Adjusted for: Socioeconomic status, perceived stress, physical activity level, over the counter analgesics, grade level, screen time and sex

### Sensitivity analyses

Adjusted sensitivity analysis using 7 h of sleep as a cut-off showed a stronger association with lower life satisfaction than 8 h of sleep for the total sample (B = 0.51 versus B = 0.31). Similar findings of stronger associations using 7 h cut-off were revealed in stratified analyses by gender, in boys (B = 0.39 versus 0.27) and girls (B = 0.60 versus B = 0.33).

## Discussion

In this study, we aimed to describe sleep duration, factors impacting sleep, consequences of insufficient sleep and satisfaction with life in Norwegian adolescents and examine possible associations between adherence to the 8-hours of sleep recommendation and satisfaction with life. Findings revealed that 73% of adolescents did not meet the recommended sleep duration of at least 8 h per night, with similar results for girls and boys. 64% reported that they felt tired at school or in activities, however more prevalent in girls than boys. Screen time had a negative impact for getting enough sleep in 62% and was more prevalent among girls than boys. Gaming disturbed sleep in 23% and was more prevalent among boys. Satisfaction with life score was 7 out of 10 for the total study sample, with somewhat higher scores for boys than girls. Adhering to the 8-hours sleep recommendation was positively associated with satisfaction with life, and there were similar findings in girls and boys. All findings remained statistically significant after adjusting for SES, perceived stress, physical activity level and OTCA use.

Our findings, revealing that 73% of the adolescents did not adhere to the 8-hours of sleep recommendation, are higher compared to international data, which shows that across countries, 32–86% of adolescents meet sleep recommendations [3]. However, not adhering to the sleep recommendation appears to be common in Norway. In a Norwegian study by Saxvig and colleagues, it was revealed that 84.8% of adolescents aged 16–17 did not adhere to the recommendation of 8-hour sleep [10]. These findings show a slightly higher prevalence compared to this current study, which may be due to several methodological differences in self-reporting. Saxvig and colleagues presents findings of sleep duration during schooldays, whereas the question provided by Ungdata refers to “how many hours did you sleep last night?”. Assuming that some Ungdata surveys were conducted on Mondays, the findings may be less comparable to data from schooldays, as adolescents commonly report a relatively large discrepancy between sleep duration on schooldays and weekends [35]. A recent Norwegian study from 2023 reported that younger Norwegian adolescents tend to sleep one and a half hours longer on weekends compared to schooldays [36]. Despite this, our findings point to the commonality of failing to obtain the recommendation of 8 h of sleep in the everyday life of Norwegian adolescents.

Estimating sleep duration by self-report in adolescence is challenging due to observed discrepancies between self-reported sleep and objectively measured sleep. However, research evidence suggests that adolescents aged 13–17 years may more precisely estimate their own sleep duration compared to when their parents report on their behalf, as parents tend to report an idealized version [37]. Objective measures, including actigraphy and the currently considered gold standard, polysomnography, offer potential clinical advantages compared to self-reporting [38]. However, these advantages are primarily related to pathological conditions, such as accurate diagnosis of sleep disorders and treatment monitoring. Lucas-Thompson and colleagues investigated the between- and within-person associations between self-reported and actigraph-measured nighttime sleep duration in adolescence [39]. The findings indicated that adolescents reporting longer average nighttime sleep also exhibited longer average actigraph measured sleep duration [39], suggesting that self-reporting in large samples of adolescence is likely to have high validity. Still, there are potential biases that should be discussed, which could be threating the validity of the study, such as self-report bias, including recall bias or social desirability bias. Despite the study is anonymous, there is no guarantee that adolescents´ didn’t under or overestimate their scores based on poor recollection or because of being afraid of observant classmates. Other relevant bias to mention is selection bias. Although the study includes the majority of Norwegian adolescents, findings may not accurately reflect the total target population.

Interestingly, our descriptive findings revealed similar sleep duration in girls and boys, which is in accordance with international data and other Norwegian sleep studies [10, 36, 40]. However, our descriptive findings revealed some differences in terms of feeling tired (sleepiness). Only 29% of girls reported they never felt sleepy during school or in activities, whereof 44% of the boys reported the same. There might be underlying mechanisms related to sleep quality or productivity differences between girls and boys that might interfere, or it could be related to other aspects of adolescents’ life, such as difference in physical activity levels and gender preferences for activities provided at schools. Nevertheless, Forest and colleagues also reported gender differences in daytime sleepiness during school and social activities in adolescents, with girls perceiving more interference from poor sleep on daytime functioning compared to boys [41]. Findings indicate other measures than sleep duration is needed for understanding daytime functioning in girls and boys. A meta-analytic review from a school setting, showed that sleepiness revealed the strongest association to school performance, followed by adolescents sleep quality and sleep duration [42].

Another gender difference was that more girls than boys reported that screen time negatively impacted their ability to sleep. It is reported that time spent in front of a screen usually comes at the expense of sleep [43]. The inability to sleep and screen time use at night are physiologically linked to the brightness and type of light, and such activity inhibit melatonin production, disrupt the circadian rhythm, and consequently affect adolescents´ feeling of sleepiness before bedtime [44]. Therefore, the systematic review by Hale et al., explicitly advises to limit or reduce screen time exposure, especially before or during bedtime hours, to minimize any harmful effects of screen time on sleep and well-being [13]. Moreover, Hale and colleagues reported that adolescents spend about 7 h per day in front of a screen [13]. Gaming might also contribute to the total screen time in adolescence. In our study, more boys than girls reported that gaming affected their ability to sleep. Time spent on video gaming in adolescence is also reported to be negatively associated with sleep duration [45].

It is interesting to link the differences in daytime sleepiness between girls and boys to the differences in satisfaction with life, as we suspect that there could be coinciding factors at play. Given that girls tend to experience more tiredness and sleepiness, it would presumably influence their subjective well-being and satisfaction with life, as sleepiness is strongly associated with adolescents’ overall quality of life [46]. Extensive research evidence has reported gender differences in health-related quality of life (HRQOL) and satisfaction with life, wherein girls tend to report lower scores than boys [24, 47–50]. Moreover, our findings of satisfaction with life align with the “Better Life index” score from the OECD, which reports 7.3 as an average score for Norwegians [22]. Interestingly, in our study, both girls and those not adhering to the sleep recommendations had coinciding satisfaction with life scores below 7.0.

As hypothesized, the findings showed that adolescents adhering to the 8-hour sleep recommendation had higher life satisfaction compared to adolescents sleeping 7 h or less. Quite similar results were found in both girls and boys, despite a slightly lower *p*-value was revealed among girls compared to boys, both associations remained significant after adjusting for relevant covariates. Indicating respective associations relevant for the total study sample. Interestingly, a Norwegian study by Ness and Saksvik-Lehouillier investigated the relationship between sleep and satisfaction with life in Norwegian university students. Their results indicated that all sleep parameters, such as sleep quality, less variability in rise time, less variability in sleep duration, longer mean sleep duration were associated with better satisfaction with life. However, less variability of sleep duration was identified as a significant predictor for life satisfaction and not mean sleep duration, indicating that less variability of sleep duration might be more relevant to well-being than sleep duration itself [51]. Research evidence also reports higher risks of negative health outcomes with higher variability in sleep duration from weekdays to weekends in adolescents [35, 52]. Further, a recent Norwegian study reported that sleep duration on weekdays was positively associated with all aspects of adolescents´ HRQOL, whereas sleep duration on weekends revealed mostly nonsignificant findings regarding aspects of HRQOL [36]. These findings highlight the vulnerability of using only one general sleep duration measure to understand the complexity between sleep and satisfaction with life. Nevertheless, our findings reinforce the importance of the 8-hours sleep recommendation for Norwegian adolescents. Sleep is a multifaceted concept, including different measures such as sleep variability, sleep quality and sleepiness, all of which can have distinct impacts on adolescents’ satisfaction with life. Therefore, it is worth exploring the possibility of sleep recommendations that encompass not only sleep duration, but also explicitly address sleep variability and daytime sleepiness in adolescence in the future.

### Strengths and limitations

The primary strength of this study lies in its large sample size, comprising adolescents from both urban and rural regions of Norway, collected within a school-based setting. Additionally, the high response rate (99%) regarding variables related to sleep and life satisfaction enhances the study’s reliability. These factors suggest that the findings could be generalizable for a broader population of Norwegian adolescents attending school. The question regarding sleep duration is based on SOT until awakening time, which is considered an accurate estimation of sleep duration [10]. Further, Ungdata dataset is cleaned and they have several procedures for identifying unserious answers [26]. Moreover, reporting according to STROBE guidelines [25] should be considered a strength, as it provides transparency and accurate reporting of study method and results.

This study also has some limitations. The cross-sectional nature of the study hinders us from determining any causal inference between sleep duration and life satisfaction. Further, another limitation is the use of non-validated instruments regarding sleep as the respective questions in Ungdata derives from an unknown origin [27]. Moreover, the sleep questions did not distinguish between weekdays and weekends, which might have affected the results. Another limitation is that the scope of this paper was focused on adhering to sleep recommendations or not, and as a result, the sleep duration variable was dichotomized. This dichotomization reduced variability in data and excluded other potential sleep-related variables that could have impacted adolescents’ satisfaction with life. Another limitation is due to study variables are measured over different time frames, as exposure is measured within last day and outcome over a few days. The predicting sleep variable would be more robust if data was provided over a longer period, which would convey a better understanding of sleep variability and average sleep duration. Moreover, we do not have any information on the non-responders, which increases the risk of selection bias. Finally, despite significant statistical associations, caution should be exercised when interpeting the findings for clinical relevance. Still, sensitivity analysis using 7 h of sleep as a cut-off shows a stronger association with lower life satisfaction than 8 h of sleep. This might indicate that less sleep is more strongly related to lower life satisfaction. This should be further explored in future studies investigating sleep as a continuous variable. However, we chose to dichotomize the variable according to sleep recommendations to make it clear and easy to interpret for adolescents, practitioners, and policymakers.

### Perspectives

This study showed that the majority of adolescents did not adhere to the 8-hours of sleep recommendation, and many of them reported feeling tired at school or in activities. Screen time and gaming were identified as descriptive factors affecting adolescent’s ability to get enough sleep. Our study added new findings to the research literature by uncovering that sleep recommendations were positively associated with higher life satisfaction by controlling for several relevant covariates in a large sample of Norwegian adolescents, underpinning essential information for people working with adolescents and caregivers. Finally, practice and policy aiming at increasing health and satisfaction with life in adolescents should include and highlight sleep recommendations.

## Conclusions

This cross-sectional study demonstrated that almost three out of four Norwegian adolescents did not meet the sleep recommendations, and close to two thirds reported that they feel tired at school or in activities. Screen time negatively affected their ability to get enough sleep. Findings revealed a positive association between adhering to the 8-hours of sleep recommendation and satisfaction with life. These findings reinforce the importance of adhering the sleep recommendation for Norwegian adolescents. Adolescence is a critical time wherein insufficient sleep can have significant consequences. Further research is needed to examine other sleep related measures to adolescents’ satisfaction with life.

### Electronic supplementary material

Below is the link to the electronic supplementary material.

Supplementary Material 1

Supplementary Material 2

## Data Availability

The dataset that support the findings of this study is available upon reasonable request from the Norwegian Agency for Shared Services in Education and Research (SIKT) [34]. Dataset citation required from SIKT: 10.18712/NSD-NSD3007-V3.

## Abbreviations

TIB: time in bed
SOT: sleep onset time
CI: confidence interval
SD: standard deviation
OR: odds ratio
SES: socioeconomic status
NOVA: Norwegian Social Research
Korus: regional center for drug rehabilitation
KS: the municipal sector´s organization
STROBE: Strengthening The Reporting Of Observational Studies
SIKT: Norwegian Agency for Shared Services in Education and Research
OECD: better policies for better lives.

## References

[1] Matricciani L (2019). Children’s sleep and health: a meta-review. Sleep Med Rev.

[2] Hirshkowitz M (2015). National Sleep Foundation’s updated sleep duration recommendations: final report. Sleep Health.

[3] Gariepy G (2020). How are adolescents sleeping? Adolescent sleep patterns and Sociodemographic Differences in 24 European and North American Countries. J Adolesc Health.

[4] Owens JA, Weiss MR (2017). Insufficient sleep in adolescents: causes and consequences. Minerva Pediatr.

[5] Lee YJ (2012). Insufficient sleep and suicidality in adolescents. Sleep.

[6] Palmer CA (2018). Associations among adolescent sleep problems, emotion regulation, and affective disorders: findings from a nationally representative sample. J Psychiatr Res.

[7] Shochat T, Cohen-Zion M, Tzischinsky O (2014). Functional consequences of inadequate sleep in adolescents: a systematic review. Sleep Med Rev.

[8] Owens J (2014). Insufficient sleep in adolescents and young adults: an update on causes and consequences. Pediatrics.

[9] Konjarski M (2018). Reciprocal relationships between daily sleep and mood: a systematic review of naturalistic prospective studies. Sleep Med Rev.

[10] Saxvig IW (2021). Sleep in older adolescents. Results from a large cross-sectional, population-based study. J Sleep Res.

[11] Thorleifsdottir B (2002). Sleep and sleep habits from childhood to young adulthood over a 10-year period. J Psychosom Res.

[12] Baiden P, Tadeo SK, Peters KE (2019). The association between excessive screen-time behaviors and insufficient sleep among adolescents: findings from the 2017 youth risk behavior surveillance system. Psychiatry Res.

[13] Hale L, Guan S (2015). Screen time and sleep among school-aged children and adolescents: a systematic literature review. Sleep Med Rev.

[14] Roeser K (2012). Relationship of sleep quality and health-related quality of life in adolescents according to self- and proxy ratings: a questionnaire survey. Front Psychiatry.

[15] Schmidt RE, Van der M, Linden (2015). The relations between Sleep, personality, behavioral problems, and School Performance in adolescents. Sleep Med Clin.

[16] Yeo SC (2019). Associations of sleep duration on school nights with self-rated health, overweight, and depression symptoms in adolescents: problems and possible solutions. Sleep Med.

[17] Gradisar M, Gardner G, Dohnt H (2011). Recent worldwide sleep patterns and problems during adolescence: a review and meta-analysis of age, region, and sleep. Sleep Med.

[18] Jakobsson M, Josefsson K, Högberg K (2020). Reasons for sleeping difficulties as perceived by adolescents: a content analysis. Scand J Caring Sci.

[19] Chaput JP (2016). Systematic review of the relationships between sleep duration and health indicators in school-aged children and youth. Appl Physiol Nutr Metab.

[20] Gustafsson ML (2016). Association between amount of sleep, daytime sleepiness and health-related quality of life in schoolchildren. J Adv Nurs.

[21] Paiva T, Gaspar T, Matos MG (2015). Sleep deprivation in adolescents: correlations with health complaints and health-related quality of life. Sleep Med.

[22] *Satisfaction with life. Accessed 03.10.23*. https://www.oecdbetterlifeindex.org/topics/life-satisfaction/

[23] Diener E (1984). Subjective well-being. Psychol Bull.

[24] Chen X et al. Gender differences in life satisfaction among children and adolescents: a Meta-analysis. J Happiness Stud, 2020. 21.

[25] von Elm E (2007). The strengthening the reporting of Observational studies in Epidemiology (STROBE) Statement: guidelines for reporting observational studies. Ann Intern Med.

[26] Frøyland LR. *Ungdata – Lokale ungdomsundersøkelser. Dokumentasjon av variablene i spørreskjemaet. NOVA. 2017*.

[27] Bakken A. *Ungdata 2021. Nasjonale resultater* 2021.

[28] 05.10. 2023, A. *Young in Oslo in 2018*. https://www.oslomet.no/forskning/forskningsprosjekter/ung-i-oslo-2018

[29] Cheung F, Lucas RE (2014). Assessing the validity of single-item life satisfaction measures: results from three large samples. Qual Life Res.

[30] Jovanović V (2016). The validity of the satisfaction with Life Scale in adolescents and a comparison with single-item life satisfaction measures: a preliminary study. Qual Life Res.

[31] Bakken A, Frøyland LR, Sletten MA. *Sosiale forskjeller i unges liv. Hva sier Ungdata-undersøkelsene?* NOVA Rapport 3/2016:, 2016.

[32] Grasaas E (2022). The relationship between stress and health-related quality of life and the mediating role of self-efficacy in Norwegian adolescents: a cross-sectional study. Health Qual Life Outcomes.

[33] Thorsén F (2020). Sleep in relation to psychiatric symptoms and perceived stress in Swedish adolescents aged 15 to 19 years. Scand J Child Adolesc Psychiatr Psychol.

[34] *Norwegian Agency for Shared Services in Education and Research (SIKT)*. Accessed 05.10.2023; https://sikt.no/en/home

[35] Kim J et al. The impact of Weekday-to-Weekend Sleep Differences on Health Outcomes among adolescent students. Child (Basel), 2022. 9(1). 10.3390/children9010052 PMC8774225 35053677

[36] Grasaas E (2023). Sleep duration in schooldays is associated with health-related quality of life in Norwegian adolescents: a cross-sectional study. BMC Pediatr.

[37] Short MA (2013). Estimating adolescent sleep patterns: parent reports versus adolescent self-report surveys, sleep diaries, and actigraphy. Nat Sci Sleep.

[38] Matthews KA (2018). Similarities and differences in estimates of sleep duration by polysomnography, actigraphy, diary, and self-reported habitual sleep in a community sample. Sleep Health.

[39] Lucas-Thompson RG, Crain TL, Brossoit RM (2021). Measuring sleep duration in adolescence: comparing subjective and objective daily methods. Sleep Health.

[40] Saelee R (2023). Racial/Ethnic and Sex/Gender Differences in Sleep Duration Trajectories from Adolescence to Adulthood in a US National Sample. Am J Epidemiol.

[41] Forest G (2022). Gender differences in the interference of sleep difficulties and daytime sleepiness on school and social activities in adolescents. Sleep Med.

[42] Dewald JF (2010). The influence of sleep quality, sleep duration and sleepiness on school performance in children and adolescents: a meta-analytic review. Sleep Med Rev.

[43] Cheung CHM (2017). Daily touchscreen use in infants and toddlers is associated with reduced sleep and delayed sleep onset. Sci Rep.

[44] Wood B (2013). Light level and duration of exposure determine the impact of self-luminous tablets on melatonin suppression. Appl Ergon.

[45] Pérez-Chada D et al. Screen use, sleep duration, daytime somnolence, and academic failure in school-aged adolescents. PLoS ONE, 2023. 18(2 February). 10.1371/journal.pone.0281379 PMC9928097 36787301

[46] Ahmadi Z, Omidvar S (2022). The quality of sleep and daytime sleepiness and their association with quality of school life and school achievement among students. J Educ Health Promot.

[47] Meade T, Dowswell E (2015). Health-related quality of life in a sample of Australian adolescents: gender and age comparison. Qual Life Res.

[48] Rabbitts JA (2016). Association between widespread Pain scores and functional impairment and health-related quality of life in clinical samples of children. J Pain.

[49] Bisegger C (2005). Health-related quality of life: gender differences in childhood and adolescence. Soz Praventivmed.

[50] Michel G (2009). Age and gender differences in health-related quality of life of children and adolescents in Europe: a multilevel analysis. Qual Life Res.

[51] Ness TEB, Saksvik-Lehouillier I. The relationships between life satisfaction and Sleep Quality, Sleep Duration and Variability of Sleep in University students. Journal of European Psychology Students; 2018.

[52] Kim SJ (2011). Relationship between weekend catch-up sleep and poor performance on attention tasks in Korean adolescents. Arch Pediatr Adolesc Med.

