# Supplementary material for: Adherence to sleep recommendations is associated with higher satisfaction with life among Norwegian adolescents
Source: BMC Public Health. 2024 May 10;24:1288. doi: 10.1186/s12889-024-18725-1 (PMC11088050; doi:10.1186/s12889-024-18725-1)
Supplement: Supplementary file 1 — Supplementary Material 1 [file 12889_2024_18725_MOESM1_ESM.docx]

Supplementary table:

**Supplementary table** Response rate in study variables

| Variable | N | Response rate % |
| --- | --- | --- |
| Sleep duration | 32,055 | 99.7% |
| Problems falling asleep | 29,741 | 92.5% |
| Being tired in school or in activities | 31,558 | 98.1% |
| Screen time affecting not getting enough sleep | 2,114 | 99.8% |
| Gaming affecting not getting enough sleep | 15,936 | 99.8% |
| Satisfaction with life  SES  Perceived stress  Physical activity  OTCA | 31,960  32,089  30,386  31,819  31,850 | 99.4%  99.9%  94.5%  98.9%  99.0% |
